# Weighted gene co-expression network analysis reveals hub genes regulating response to salt stress in peanut

**DOI:** 10.1186/s12870-024-05145-x

**Published:** 2024-05-20

**Authors:** Feifei Wang, Huarong Miao, Shengzhong Zhang, Xiaohui Hu, Ye Chu, Weiqiang Yang, Heng Wang, Jingshan Wang, Shihua Shan, Jing Chen

**Affiliations:** 1 Shandong Peanut Research Institute, Qingdao, 266100 People’s Republic of China; 2 Department of Horticulture, University of Georgia Tifton Campus, Tifton, GA 31793 USA; 3 Agricultural Technical Service Center, Rizhao, 276700 Shandong China; 4 College of Agronomy, Qingdao Agricultural University, Qingdao, 266109 People’s Republic of China

**Keywords:** Peanut, Salt tolerance, Root, Shoot, WGCNA, Hub genes

## Abstract

Peanut (*Arachis hypogaea* L.) is an important oilseed crop worldwide. However, soil salinization becomes one of the main limiting factors of peanut production. Therefore, developing salt-tolerant varieties and understanding the molecular mechanisms of salt tolerance is important to protect peanut yield in saline areas. In this study, we selected four peanut varieties with contrasting response to salt challenges with T1 and T2 being tolerance and S1 and S2 being susceptible. High-throughput RNA sequencing resulted in more than 314.63 Gb of clean data from 48 samples. We identified 12,057 new genes, 7,971of which have functional annotations. KEGG pathway enrichment analysis of uniquely expressed genes in salt-tolerant peanut revealed that upregulated genes in the root are involved in the MAPK signaling pathway, fatty acid degradation, glycolysis/gluconeogenesis, and upregulated genes in the shoot were involved in plant hormone signal transduction and the MAPK signaling pathway. Na^+^ content, K^+^ content, K^+^/ Na^+^, and dry mass were measured in root and shoot tissues, and two gene co-expression networks were constructed based on weighted gene co-expression network analysis (WGCNA) in root and shoot. In this study, four key modules that are highly related to peanut salt tolerance in root and shoot were identified, plant hormone signal transduction, phenylpropanoid biosynthesis, starch and sucrose metabolism, flavonoid biosynthesis, carbon metabolism were identified as the key biological processes and metabolic pathways for improving peanut salt tolerance. The hub genes include genes encoding ion transport (such as *HAK8*, *CNGCs*, *NHX*, *NCL1*) protein, aquaporin protein, CIPK11 (CBL-interacting serine/threonine-protein kinase 11), LEA5 (late embryogenesis abundant protein), POD3 (peroxidase 3), transcription factor, and MAPKKK3. There were some new salt-tolerant genes identified in peanut, including cytochrome P450, vinorine synthase, sugar transport protein 13, NPF 4.5, IAA14, zinc finger CCCH domain-containing protein 62, beta-amylase, fatty acyl-CoA reductase 3, MLO-like protein 6, G-type lectin S-receptor-like serine/threonine-protein kinase, and kinesin-like protein KIN-7B. The identification of key modules, biological pathways, and hub genes in this study enhances our understanding of the molecular mechanisms underlying salt tolerance in peanuts. This knowledge lays a theoretical foundation for improving and innovating salt-tolerant peanut germplasm.

**Supplementary Information:**

The online version contains supplementary material available at 10.1186/s12870-024-05145-x.

## Introduction

Salt stress is a prevalent abiotic factor, affecting over 1000 million hectares, or approximately 6–7% of the world’s total land area [1]. Saline soil, distinguished by an elevated salts concentration, mainly from sodium chloride (NaCl), poses a significant challenge to for crop production. Generally, glycophytes are under stress when expose to an osmotic pressure of -0.2 MPa caused by 40 mmol L^−^ of NaCl in the soil [2, 3]. Accordingly, 45 Mha out of the total 230 Mha of global irrigated farmland is considered high in soil salinity [3–5]. The increase in acreage of saline farmland is worsened by the combined impacts of global climate change, excessive fertilization, and irrigation [6, 7]. This trend is notably pronounced in arid and semiarid regions. Many crops such as maize, rice, wheat, soybean, and peanut are glycophytes that tolerate only low salt or mild salt stress [6–8]. The growth of most crops undergoes a significant 25% decline when exposed to a concentration of 50 mmol L^−1^ NaCl. Furthermore, salt concentration of 100 mmol L^−1^ NaCl leads to substantial impairment in most crop production and, in some cases, complete crop failure [2, 3, 5]. Peanut (*Arachis hypogaea* L.) is an oilseed crop with a planting area of nearly 30 Mha worldwide and yielded more than 50 million tons in 2023 (USDA, https://www.usda.gov). China is the third-largest country covered by saline-alkali land in the world [9]. It is urgent to develop salt-tolerant peanut varieties and uncover the regulatory mechanism of salt response in peanut.

Salt imposes stress to crops through osmotic and ionic pressure. Osmotic stress in root begins immediately upon exposure to a high salt concentration, while ionic stress occurs in leaves upon excessive accumulation of Na^+^ and Cl^−^ [6]. Plants have evolved intricate pathways to withstand the adverse effects of environmental stress. These pathways regulate osmotic and oxidative stresses, transport and compartmentalize salt ions, and manage the trade-off between growth and salt tolerance [6]. The osmotic signal triggers the accumulation of abscisic acid (ABA), which activates the ABA signaling pathway. This activation leads to a decrease in turgor pressure, induces stomatal closure, accumulates osmolytes, facilitates water and nutrient uptake [10, 11]. Stress responsive genes such as *β*‐amylase1, *α*‐amylase, CCCH zinc finger protein, and late embryogenesis abundant protein (LEA) have been reported to be involved in the regulation of osmotic stress [12–14]. Mitogen‐activated protein kinase cascades (MAPK) signals also play important roles in response to osmotic stress of plants [15].

Under salt stress, excess sodium (Na^+^) in the roots is managed through three pathways: exportation from the root to soil solutions, sequestration into vacuoles, and transportation to the shoot. The SOS pathway, comprising SOS1 (Na^+^/H^+^ antiporter), SOS2 (also known as CBL‐interacting protein kinase 24 or CIPK24), and SOS3-like calcium-binding protein 8 (SCaBP8, also called calcineurin b‐like protein10 or CBL10), serves as a crucial mechanism for mediating the export of Na + ions from the roots [16]. For many plants, the primary site of Na + toxicity is the leaves-where photosynthesis takes place. Na^+^ remaining in the root is sequestered into vacuoles or transported to the shoot. The compartmentalization of Na^+^ into vacuoles is facilitated by vacuolar Na^+^/H^+^ exchangers (NHXs) [17]. Plants employ various mechanisms to limit the loading of Na^+^ into the root xylem and enhance the retrieval of Na + from the root xylem, thereby reducing the translocation of Na^+^ from roots to shoots. HKT (high-affinity K^+^ transporter) members play pivotal roles in this process [6].

When cytoplasmic Na + levels rise, the membrane potential drops below the resting level, activating K^+^ outflow channels and disrupting the balance between K^+^ and Na^+^ homeostasis [18]. The K^+^ transporter/high-affinity K^+^ transporter/K^+^ uptake protein (KT/HAK/KUP) family serves as a key K^+^ acquisition system in plants, regulating K^+^ uptake and translocation [19]. Chloride (Cl^−^) serves as an essential nutritional element, playing pivotal roles in stomatal movement, maintenance of cell turgor, and photosynthesis [20, 21]. Excessive accumulation of chloride ions (Cl^−^) can impede the uptake, transport, and assimilation of nitrate ions (NO_3_^−^), ultimately result in chloride toxicity. Several transporters capable of facilitating chloride transport have been identified, including nitrate transporter/peptide transporters (NPFs) and chloride channels (CLCs) [6, 22]. In addition to the effects on ion balance, salt stress can induce the accumulation of reactive oxygen species (ROS) and oxidative stress. The main ROS in plants include hydroxyl radicals, hydrogen peroxide (H_2_O_2_), superoxide anions, and singlet oxygen. Excessive ROS accumulation can damage cells through processes such as lipid peroxidation in cellular membranes, DNA damage, protein denaturation, oxidation of carbohydrates, breakdown of pigments, and impairment of enzymatic activities. To counteract the harmful effects of ROS, plants utilize a network of antioxidant enzymes including superoxide dismutase (SOD), catalase (CAT), ascorbic acid peroxidase (APX), glutathione peroxidase (GPX), glutathione reductase (GR), dehydroascorbate reductase (DHAR), monodehydroascorbate reductase (MDHAR), and glutathione S-transferase (GST). These enzymes work cooperatively to scavenge ROS and maintain cellular redox homeostasis under salt stress [23, 24].

High-throughput RNA sequencing (RNA-seq) has been used to decipher the physiological, biochemical, and molecular adaptations related to plant response to stresses. Weighted gene co-expression network analysis (WGCNA) is a molecular biology network analysis method widely utilized in the study of crops response to abiotic stress [25], including cotton (*Gossypium hirsutum* L.) [26, 27], maize (*Zea mays* L.) [28, 29], rice (*Oryza sativa* L.) [30], wheat (*Triticum aestivum* L.) [31], and tomato (*Solanum lycopersicum* L.) [32]. These studies reported regulatory pathways and key genes associated with salt adaptation in plants.

In this study, we carried out WGCNA based on a gene dataset from RNA-Seq and physiological data to explore the key functional modules and biological metabolic pathways of peanut root and shoot tissue adaptation to salt stress, identify the salt-tolerant hub genes, understand the molecular mechanisms of peanut resistance to salt stress, and provide insights for the breeding of salt-resilient peanut varieties.

## Methods

### Plant materials and treatments

**Fig. 1:**
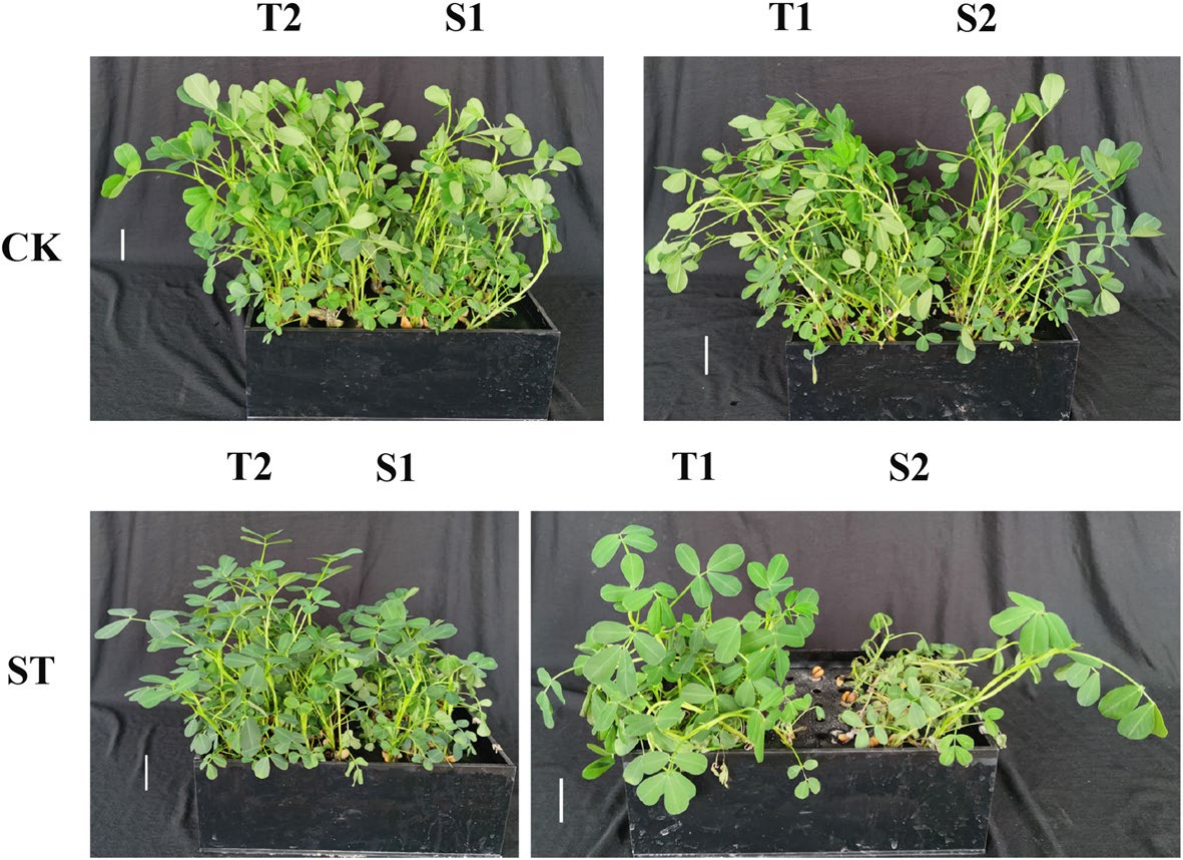
Phenotypic difference of tolerant cultivars (T1 and T2) and sensitive cultivars (S1 and S2) under salt stress. T1, T2, S1, and S2 represented Yuhua18, Huayu9510, Puhua76, and Fenhua8, respectively

**Fig. 2:**
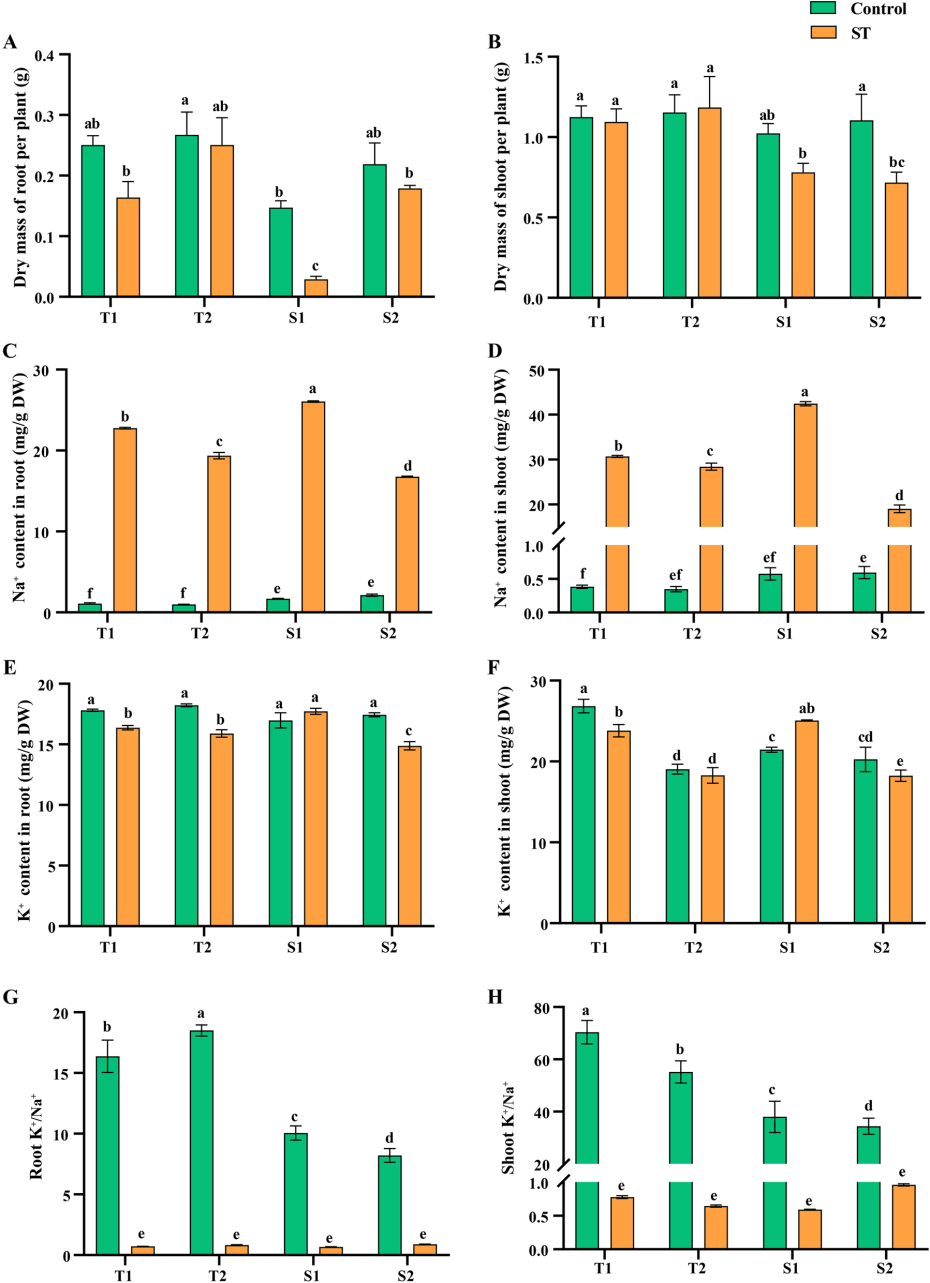
Physiological index of tolerant cultivars and sensitive cultivars under salt stress. Dry mass per plant of shoot (**A**) and root (**B**), Na^+^ content of shoot (**C**) and roots (**D**), K^+^ contents of shoot (**E**) and roots (**F**), K^+^/Na^+^ of shoot (**G**) and roots (**H**). T1, T2, S1, and S2 represented Yuhua18, Huayu9510, Puhua76, and Fenhua8, respectively. Different lowercase letters indicate a significant difference at *p* ≤ 0.05 (Duncan’s multiple range test)

Two salt-tolerant peanut varieties ("Yuhua18" and "Huayu9510") and two salt-sensitive varieties ("Fenhua8" and "Puhua76") were chosen based on their physiological and yield characteristics (Table S1, Figs. 1 and 2). The salt-tolerant varieties were developed by the College of Agronomy, Qingdao Agricultural University, and the Shandong Peanut Research Institute, respectively. The salt-sensitive varieties were released by the Industrial Crop Research Institute, Shanxi Agricultural University, and the Puyang Academy of Agricultural Sciences. Upon germination on filter papers in petri dishes for three days, peanut seedlings were transplanted into hydroponic pots filled with Hoagland's nutrient solution. They were then cultivated in an artificial climate incubator under a 16/8 light/dark cycle (200 μmol protons m^−2^ s^−1^, 28 °C) with 50% relative humidity. The nutrient solution was refreshed weekly. After two weeks, the peanut seedlings were treated with 1% NaCl solution in Hoagland's nutrient solution for seven days. The salt solution was replaced every two days. Each sample had 15 plants, and three biological replicates were performed for each sample. After treatment, shoots and roots were harvested. A portion of the samples was frozen in liquid nitrogen and stored at -80 °C for subsequent transcriptomic library construction, and another portion was used to measure K^+^ and Na^+^ concentration. The remaining shoots and roots were oven-dried at 80 °C for 72 h to obtain dry weights.

### Tissue ion analysis

The dried shoot and root samples were ground into a fine powder. Tissue samples were then extracted in a solution containing 10 ml of nitric acid (HNO_3_) and perchloric acid (HClO_4_) overnight at room temperature. Diluted samples of the extracts were subsequently analyzed for sodium ions (Na^+^) and potassium ions (K^+^) using an inductively coupled plasma emission spectrometer (ICP-OES) (iCAP 7200 HS Duo, ThermoFisher, USA) [33].

### RNA extraction and transcriptome sequencing

For each treatment (control and salt stress) and each genotype (two tolerant and two sensitive), three biological replicates were utilized for RNA-seq analysis. Total RNA was extracted from the shoot and root samples of 48 individual samples using the RNeasy Plant Mini Kit (Qiagen), following the manufacturer’s protocol. The quantity and quality of the extracted RNA were evaluated using a Nanodrop 2000 Spectrophotometer (NanoDrop Technologies, Wilmington, USA) and an Agilent 2100 BioAnalyzer (Agilent Technologies Inc., Santa Clara, CA, USA). RNA-seq libraries were constructed as follows: mRNA was isolated using magnetic beads with Oligo (dT), followed by fragmentation of the mRNA into short fragments using a fragmentation buffer. Subsequently, cDNA was synthesized using the fragmented mRNA as templates. The resulting short cDNA fragments were purified and subjected to end reparation and single nucleotide A (adenine) addition. Finally, adapters were ligated to the A-tailed cDNA fragments. The final cDNA libraries were obtained through PCR enrichment and were accurately quantified using quantitative PCR (qPCR), ensuring that the effective concentration of each library exceeded 2 nM. Subsequently, the libraries were subjected to sequencing using the Illumina Novaseq PE150 platform at Biomarker Technology Co., Ltd. (Beijing, China). Following sequencing, raw reads were acquired and processed to obtain clean reads. Raw reads were cleaned by removing adapters and unknown nucleotides. Bioinformatics analysis was conducted using the BMC Cloud platform (www.biocloud.net). High-quality sequences were then aligned to the Tifrunner reference genome (https://data.legumeinfo.org/Arachis/hypogaea/genomes/Tifrunner.gnm2.J5K5/) [34] using Hisat2 (v2.0.5) to determine the physical locations of the reads. Subsequently, StringTie was used to assemble these reads and reconstruct the transcriptome. The raw data obtained from this experiment has been deposited in NCBI.

### Differential expression analysis and gene enrichment analysis

Differential expression analysis comparing two distinct combinations was executed utilizing DESeq software (version 1.20.0). The screening criteria for identifying significantly differentially expressed genes included an expression fold change magnitude of | log_2_FoldChange |> 1, with False discovery rate (FDR) corrections of *p*-values defined at *p* < 0.05 [35]. The DEGs were obtained from two dimensions, ST vs CK of each variety tissue and T vs S in salt treated varieties tissues.

All genes were annotated by NCBI Non-Redundant Protein Sequences (NR), Homology Clusters of Proteins (KOG/COG/eggNOG), Kyoto Encyclopedia of Genes and Genomes (KEGG) and Gene Ontology (GO) database. The Gene Ontology (GO) enrichment analysis and KEGG pathway analysis were conducted using the DAVID functional annotation tool (https://david.ncifcrf.gov/home.jsp).

### Construction of the weight gene co-expression network

Genes with FPKM greater than 1 of the samples were selected for Weighted Gene Co-expression Network Analysis (WGCNA) to explore the complex relationship between genes and phenotypes [25] (www.biomark.net). For high reliability of the results, the minimum number of genes was set to 30. Genes with a correlation coefficient greater than 0.70 between modules were classified as the same module. The correlation between ME and traits was used to estimate module trait associations. Gene significance (GS) and module membership (MM) were the priorities to screen for important genes within the module. Finally, the networks of ten hub genes and top ten genes with highest connectivity were visualized using Cytoscape [36].

### Validation of the DEGs by qRT-PCR

Twelve highly expressed hub genes were screened for qRT-PCR validation. Total RNA was extracted from the same samples that were used for sequencing. First-strand cDNA was synthesized using a *Evo M-MLV* kit for qPCR (AG11705, Accurate Biotechnology(Hunan) Co., Ltd, ChangSha, China). The primer sequences used were designed with Primer 3 and synthesized by Tsingke Biotechnology (Beijing) Co., Ltd. The detail of primers was shown in the Table S4. RT-qPCR was performed on an ABI 7500 thermocyler (Applied Biosystems) using SYBR Green *Pro Taq* HS Mix (AG11701, Accurate Biotechnology(Hunan) Co., Ltd, ChangSha, China) according to the instructions. The peanut *ADH3* gene was used as the endogenous control. Gene expression levels were calculated from the threshold cycle according to 2^−ΔΔCT^ [37] and standard deviation was calculated between three biological replicates.

### Statistical analysis

Data presented are the means ± SE. The data analysis was conducted using one-way analysis of variance (ANOVA) followed by Duncan’s multiple intervals (*p* ≤ 0.05), utilizing SPSS 20.0 software (SPSS Inc., Chicago, USA). Graphs and correlation analysis of qRT-PCR and FPKM were generated using GraphPad Prism 9.0 (GraphPad Software, Inc.).

## Results

### Phenotypic difference of four peanut varieties in response to salt stress

Salt stress had minimal impact on the growth of salt-tolerant varieties (T1 and T2), whereas it significantly reduced the growth of salt-sensitive varieties (S1 and S2). Furthermore, S2 exhibited wilting in response to a 1% NaCl treatment (Fig. 1). The dry mass of both root and shoot in salt-sensitive varieties was significantly lower than the salt-tolerant varieties (Fig. 2A, B).

The salt treatment resulted in a sharp increase in shoot and root Na^+^ concentrations (measured on a tissue dry mass basis) compared with controls. Specifically, the Na^+^ concentrations in both root and shoot of the salt-sensitive variety Fenhua8 were higher than those of the salt-tolerant varieties under both control and salt treatment conditions (Fig. 2C, D). Moreover, salt treatment decreased the K^+^ concentrations of root in T1, T2, and S2 varieties and shoot in T1 and S2 varieties (Fig. 2E, F). Under normal conditions, the salt-tolerant varieties displayed notably higher K^+^/Na^+^ ratios in both root and shoot compared with the salt-sensitive varieties. However, all four genotypes exhibited similar ratios under salt treatment conditions (Fig. 2G, H).

### RNA sequencing and identification of DEGs

RNA-seq analysis of 48 samples (4 genotypes × 2 tissues × 2 treatments × 3 biological replicates) yielded a total of 314.63 Gb of clean data. The qualified clean reads were aligned to the peanut reference genome (*Arachis hypogaea*. Tifrunner. gnm2.ann1.4K0L.genome.fa), with alignment rates ranging from 87.68% to 97.42% across libraries (Table S2). The number of reads for each sample ranged from 19.08 million to 29.52 million. Among these filtered reads, > 91.71% had base quality > Q30 (Table S3).

**Fig. 3:**
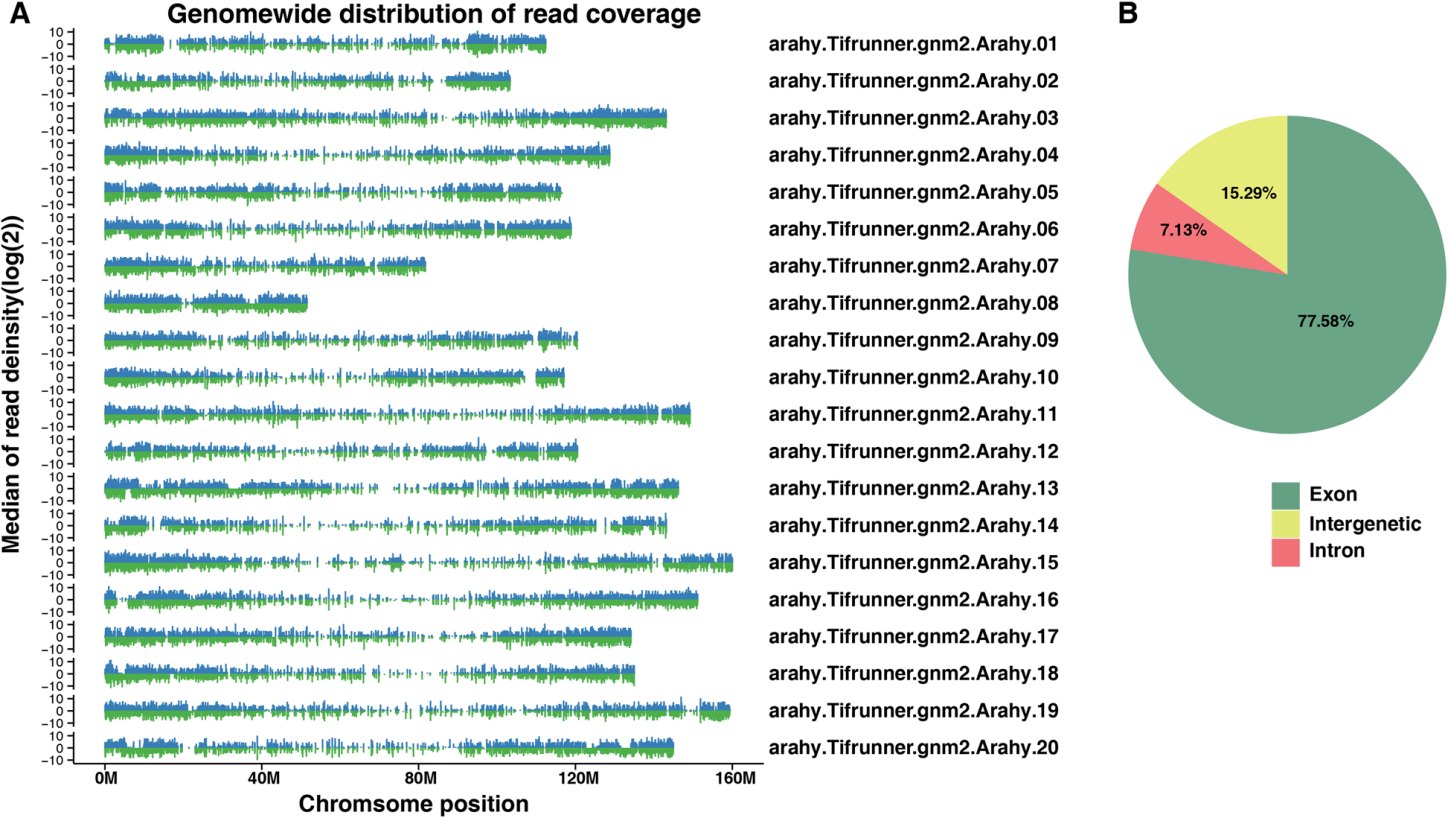
Whole genome coverage (**A**) and gene read distributions (**B**) for CK-T1-R1

Genome wide distribution analysis of the RNA-seq libraries indicates an even distribution across the reference genome for all libraries (Fig. 3A). In addition, 79.21% of the reads from our libraries mapped to the exon region (Fig. 3B). We identified 12,057 new genes that were further blasted against other annotation databases, including GO, COG, eggNOG, KEGG, KOG, Pfam, TrEMBL, NR, and Swiss-Prot. Among them, 7,971 genes that had at least one positive hit (Table S5).

**Fig. 4:**
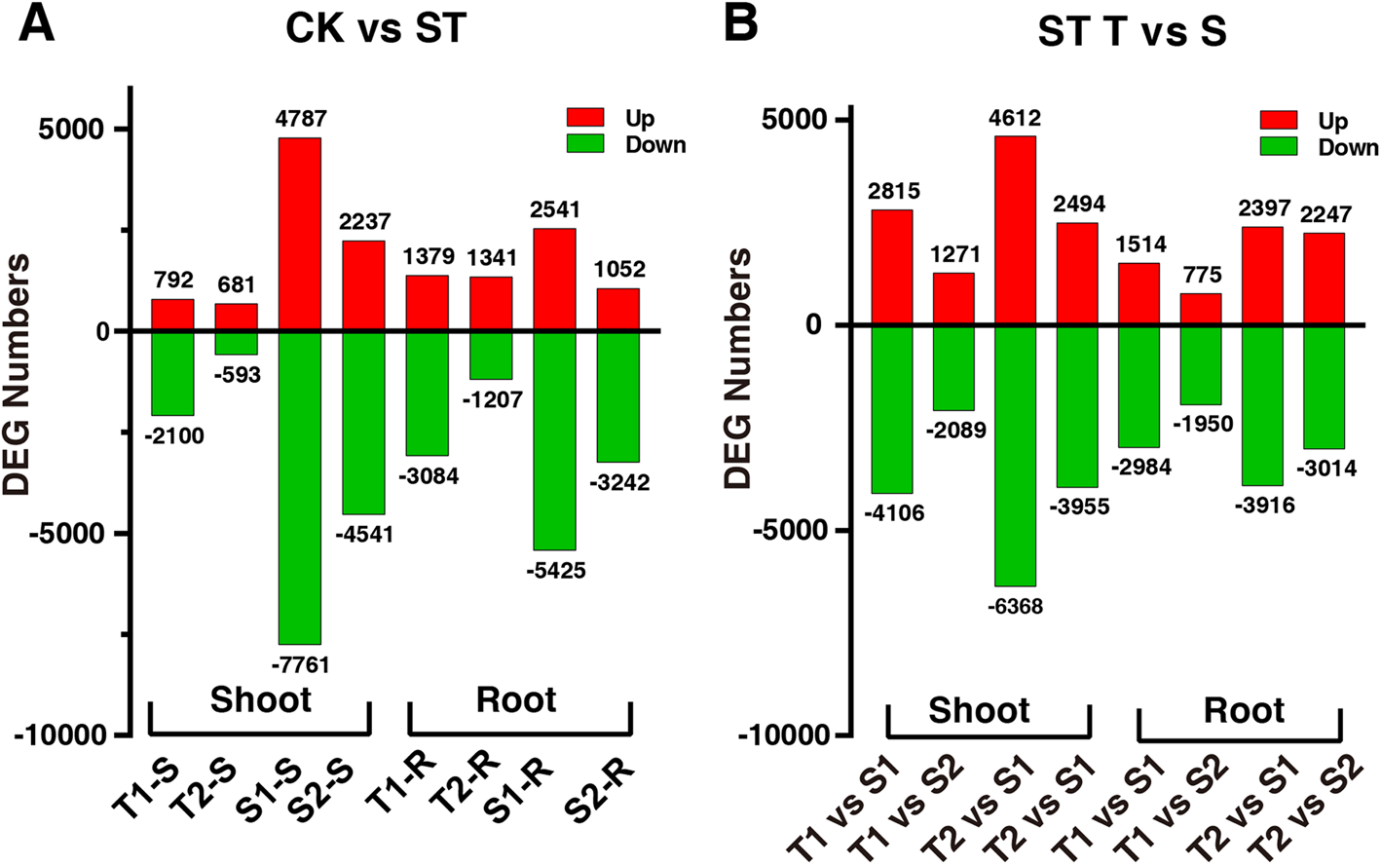
Salt induced DEGs in shoot and root of different peanut cultivars (**A**), and DEGs of tolerant and sensitive peanut cultivars in shoot and root under salt stress (**B**). T1, T2, S1, and S2 represented Yuhua18, Huayu9510, Puhua76, and Fenhua8, respectively

A total of 1379 (3084), 1341 (1207), 2541 (5425), 1052 (3242), upregulated (downregulated) DEGs in root of T1, T2, S1, S2 were screened respectively. In shoot of T1, T2, S1, S2, a total of 792 (2100), 681 (593), 4787 (7761), 2237 (4541), upregulated (downregulated) DEGs were screened respectively (Fig. 4A). Meanwhile, a total of 2815 (4106), 1271(2089), 4612 (6368), 2494 (3955), upregulated (downregulated) DEGs in salt treated root of T1 vs S1, T1 vs S2, T2 vs S1, T2 vs S2 were identified respectively. The corresponding upregulated (downregulated) DEGs in salt treated shoot were 1514 (2984), 775 (1950), 2397 (3916), 2247 (3014), respectively (Fig. 4B). Upregulated DEGs in shoot of T1 and T2 were more than that of S1 and S2 and the upregulated DEGs in root of T1 and T2 were more than that in shoot, while the opposite was true for S1 and S2 (Fig. 4A).

### DEGs upregulated uniquely in peanut salt-tolerant varieties

**Fig. 5:**
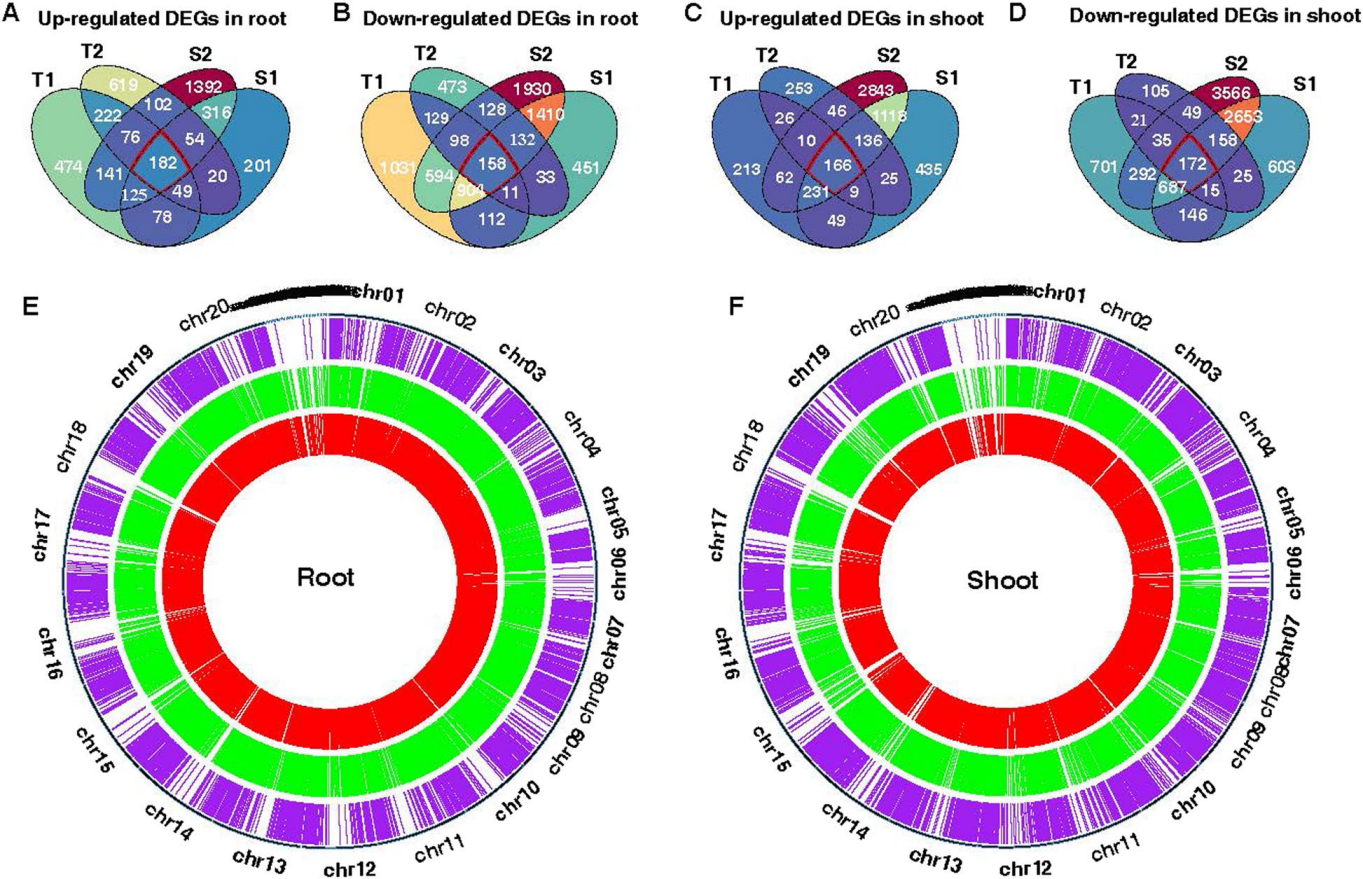
Common and unique salt-responsive DEGs in tolerant and sensitive peanut cultivars in shoot and root under salt stress. **A** Up regulated DEGs in root, **B** Down regulated DEGs in root, **C** Up regulated DEGs in shoot, **D** Down regulated DEGs in shoot. T1, T2, S1, and S2 represented Yuhua18, Huayu9510, Puhua76, and Fenhua 8, respectively. **E**–**F** Circos plot showing chromosomes (Ch1-Ch20) and scaffolds (see genome description for details) in the outermost circle, purple lines represent the salt-induced DEGs unique to salt tolerant varieties, green lines represent the salt-induced DEGs unique to salt sensitive varieties, and red lines represent DEGs between varieties with significant difference in salt tolerance

**Fig. 6:**
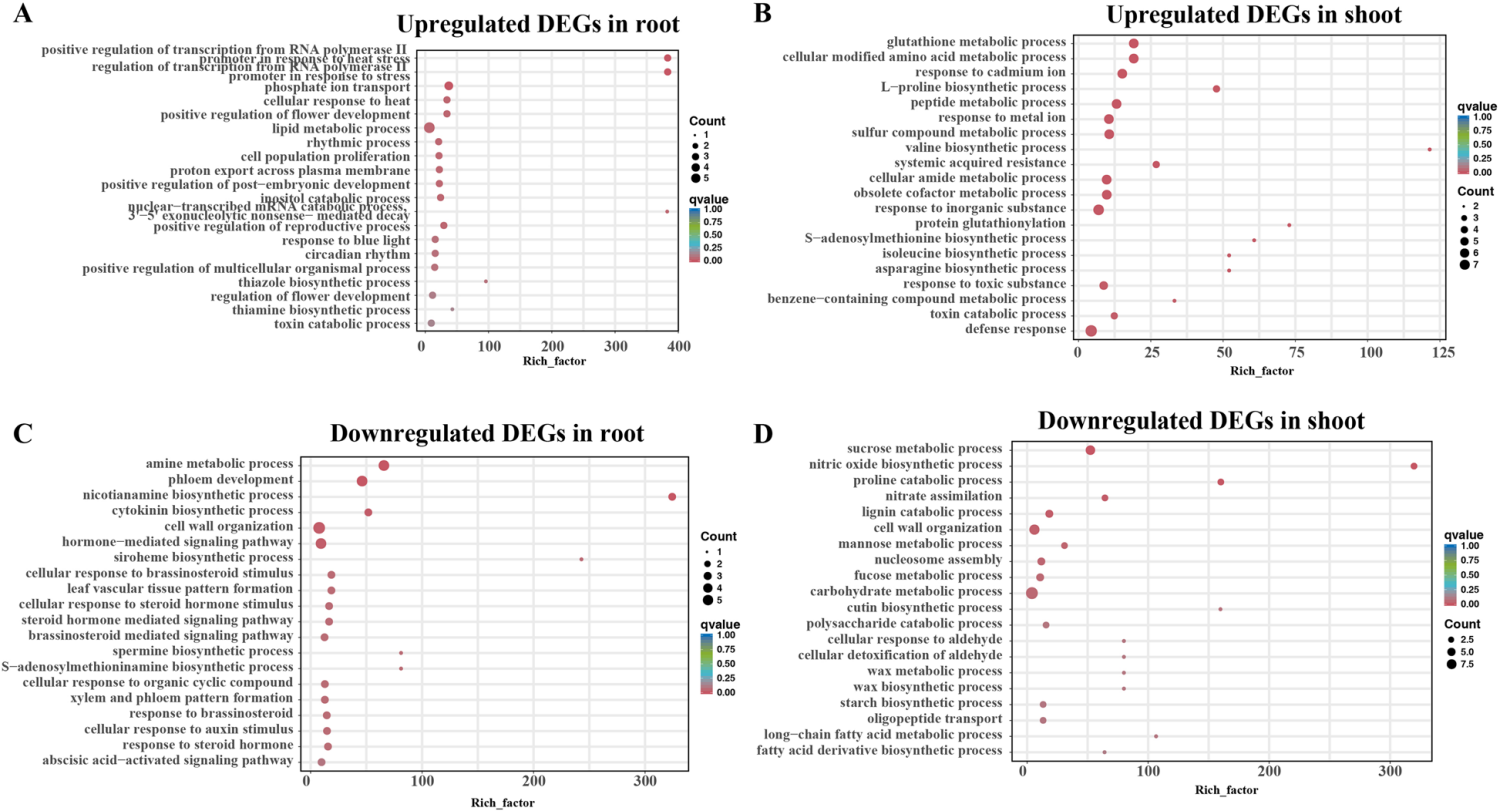
GO enrichment of common salt responsive DEGs of T1, T2, S1, and S2 in shoot and root (**A**-**D**). Rich factor, the ratio of the number of genes enriched to each GO term to the total number of genes analyzed by GO. The biological process meeting the condition of *q* value ≤ 0.05 were defined as significantly enriched. The size of circle represents gene numbers, and the color of circle represents *q* value

**Fig. 7:**
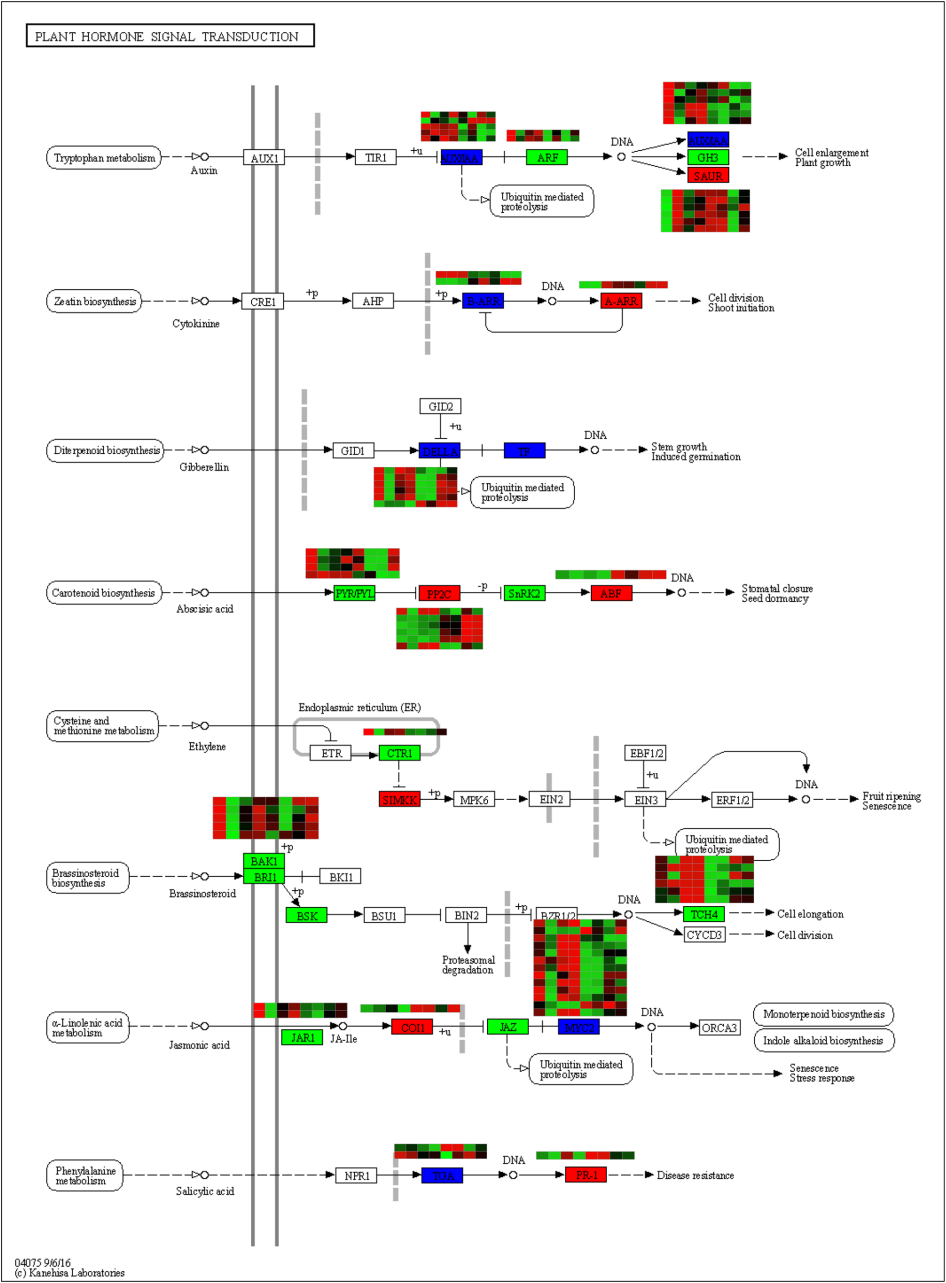
Plant hormone signal transduction detected in response to salt stress in peanut shoots. This figure provides only a KEGG pathway for up and down-regulated genes in the two salt-tolerant genotype and salt-sensitive cultivar. The heatmap represent CK-T1-S, CK-T2-S, CK-S1-S, CK-S2-S, ST-T1-S, ST-T2-S, ST-S1-S, ST -S2-S, respectively. Red and green color boxes represent the up-regulated, down-regulated genes in T1, respectively. And blue color boxes represent both up-regulated and down-regulated genes in T1

Venn diagrams were employed to illustrate the transcriptomic changes associated with salt stress across four genotypes, each displaying distinct phenotypes. In the roots, a total of 182 upregulated (158 downregulated) differentially expressed genes (DEGs) overlapped, while in the shoots, 166 upregulated (172 downregulated) DEGs were identified as common among the genotypes (Fig. 5A-D). The largest number of DEGs were distributed in chromosome 3 and 13 (Fig. 5E–F). Functional annotation and GO (gene ontology) enrichment analysis revealed that the biological processes of peanut shoot’s upregulated genes were involved in defense response and glutathione metabolic process (Fig. 6A). The peanut root’s upregulated genes were involved in lipid metabolic process, phosphate ion transport, and regulation of transcription from RNA polymerase II promoter in response to stress (Fig. 6B). GO enrichment analysis revealed that among the common downregulated DEGs in both shoots and roots, enrichment was observed in several biological processes. These included carbohydrate metabolic process and sucrose metabolic process in shoots, while in roots, enrichment was observed in cell wall organization, hormone-mediated signaling pathway, and phloem development (Fig. 6C-D). Plant hormone signal transduction pathways play pivotal roles in mediating the response to salt stress, particularly in peanut plants. The key salt-responsive genes involved in plant hormone signal transduction pathways are illustrated in Fig. 7.

**Fig. 8:**
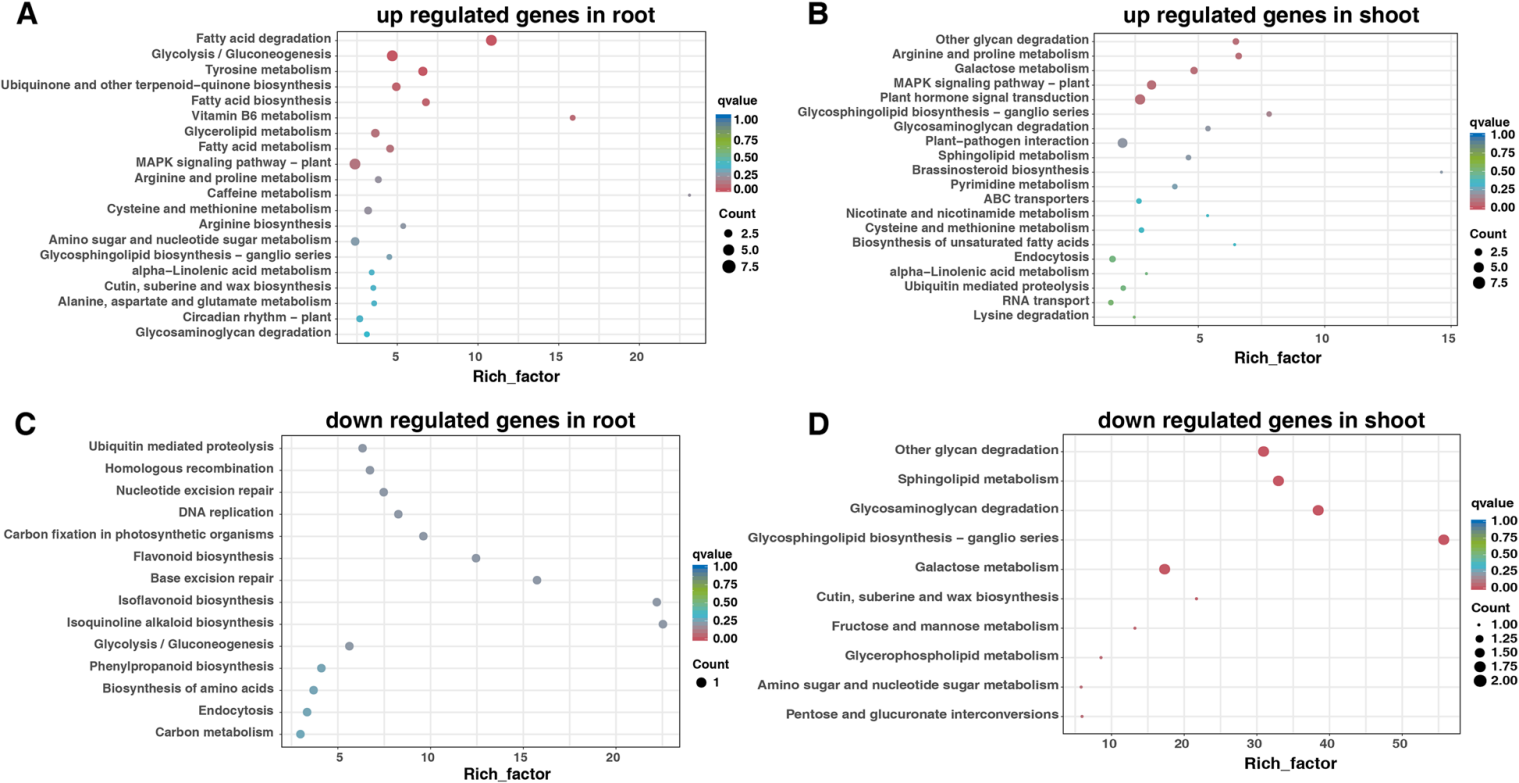
KEGG enrichment analysis of unique expressed genes in salt-tolerant varieties. **A** up regulated genes in root, **B** up regulated genes in shoot, **C** down regulated genes in root, **D** down regulated genes in shoot

In the root and shoot of salt-tolerant varieties, a total of 222 up-regulated (129 down-regulated) DEGs were uniquely identified in the root, while 26 up-regulated (21 down-regulated) DEGs were uniquely identified in the shoot (Fig. 5A-D). The KEGG pathway enrichment analysis demonstrated distinct patterns of involvement for upregulated genes in the root and shoot under salt stress. In the root, upregulated genes were notably associated with the MAPK signaling pathway, fatty acid degradation, and glycolysis/gluconeogenesis (Fig. 8A). Conversely, in the shoot, upregulated genes were predominantly associated with plant hormone signal transduction and the MAPK signaling pathway (Fig. 8B). The downregulated genes in root were associated with ubiquitin mediated proteolysis, phenylpropanoid biosynthesis, carbon metabolism, however, in the shoot it exhibited enrichment in several metabolic pathways, specifically galactose metabolism, glycosaminoglycan degradation, sphingolipid metabolism, and other glycan degradation pathways (Fig. 8C-D).

### Weighted Gene Co-expression Network Analysis (WGCNA) of root tissue response to salt stress

**Fig. 9:**
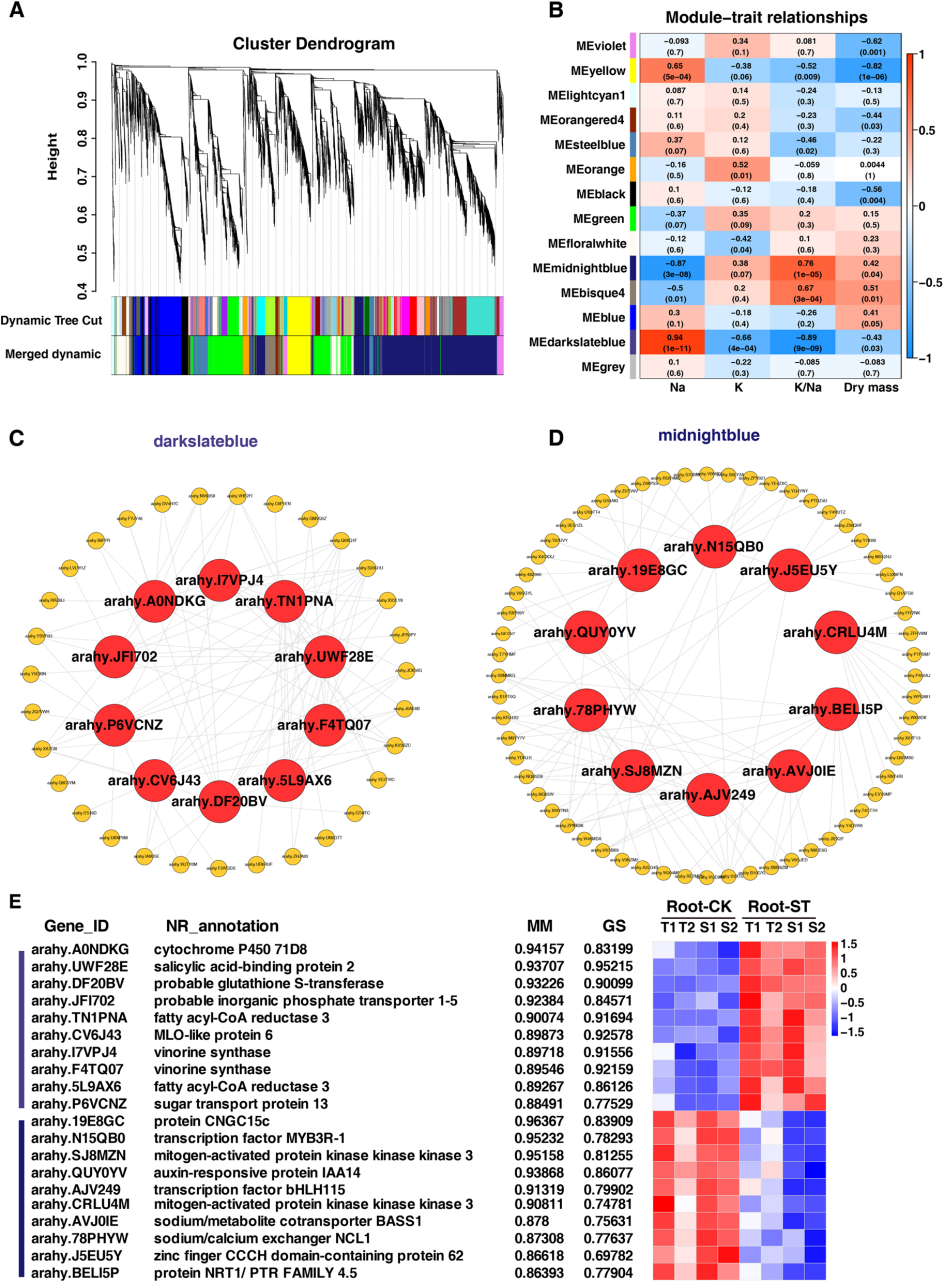
WGCNA analysis of root tissue with salt tolerance. **A** Cluster dendrograms and module division in root tissue. **B** Module-trait relationships with ion content and dry mass in root tissue with salt tolerance. The number represents the correlation coefficient about modules with traits. The red module was positive correlation, the blue was negative correlation, and the number was *p*-value. **C**-**D** Co-expression network of hub genes and top ten genes with highest connectivity for dark slate blue and midnight blue modules. **E** The expression of hub genes in dark slate blue and midnight blue modules of root tissue (*p*-value < 0.05). MM, module membership; GS, gene significance

**Fig. 10:**
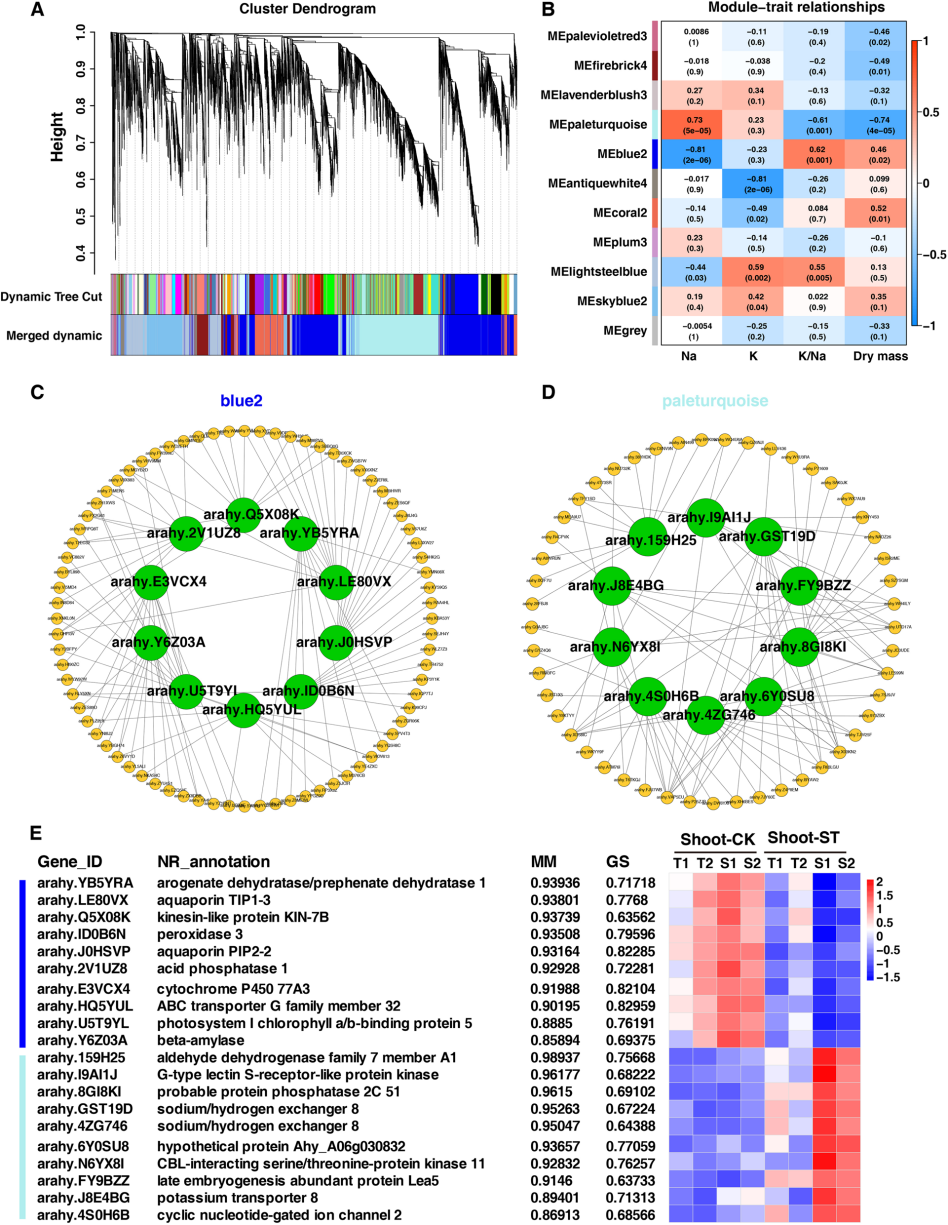
WGCNA analysis of shoot tissue with salt tolerance. **A** Cluster dendrograms and module division in shoot tissue. **B** Module-trait relationships with ion content and dry mass in root tissue with salt tolerance. The number represents the correlation coefficient about modules with traits. The red module was positive correlation, the blue was negative correlation, and the number was *p*-value. **C**-**D** Co-expression network of hub genes and top ten genes with high connectivity for blue2 and pale turquoise modules. **E** The expression of hub genes in blue2 and pale turquoise modules of root tissue (*p*-value < 0.05). MM, module membership; GS, gene significance

To further explore specific genes linked to salt tolerance in peanut root and shoot tissues, we conducted Weighted Gene Co-expression Network Analysis (WGCNA). In root tissue, we identified 14 co-expression modules comprising 8,234 genes (Fig. 10A). Notably, the dark slate blue module (containing 111 genes) showed a positive correlation with Na^+^ content but a negative correlation with K^+^ content, K^+^/ Na^+^ ratio, and dry mass, with correlation coefficients ranging from 0.43 to 0.89. Conversely, the midnight blue module (consisting of 3,109 genes) exhibited a negative correlation with Na^+^ content but a positive correlation with K^+^ content, K^+^/ Na^+^ ratio, and dry mass (Fig. 9B).

In order to identify the key genes associated with salt tolerance within the dark slate blue and midnight blue modules, gene network analysis was performed using CYTOSCAPE software. Specifically, the first 1,500 edges were considered for analysis. Following the elimination of unknown genes, the top ten genes with the highest module membership (MM) and annotations associated with abiotic stress resistance within each module were identified as “hub genes”. Subsequently, networks for each module were constructed utilizing these ten hub genes along with their top ten genes exhibiting the highest connectivity. The hub genes were visually represented as red nodes within the network (Fig. 9C-D).

The hub genes within the dark slate blue module were consistently up-regulated in root tissue and exhibited highest expression levels in T1. Conversely, the hub genes in the midnight blue module displayed a consistent down-regulation pattern in root tissue and demonstrated nearly lowest expression levels in both S1 and S2 samples (Fig. 9E). In the dark slate blue module, *arahy.A0NDKG* encodes cytochrome P450, *arahy.DF20BV* encodes glutathione S-transferase, *arahy.TN1PNA* and *arahy.5L9AX6* encode fatty acyl-CoA reductase 3 (FAR). FAR catalyzes the reduction of fatty acyl-CoA or fatty acyl carrier protein substrates, leading to the formation of primary alcohols, and can also reduce VLFA-CoA to aldehydes and participate in wax synthesis. Two genes (*arahy.I7VPJ4* and *arahy.F4TQ07*) encode vinorine synthase, an acetyltransferase. *Arahy.CV6J43* encodes MLO-like protein, a plant-specific seven-transmembrane domain protein. A sugar transport protein (STP, *arahy.P6VCNZ*) and a phosphate transporter (*arahy.JFI702*) were also identified. A phosphate transporter PHT4;6 facilitates the selective transport of Pi, and functions in protein *N*-glycosylation and cell wall biosynthesis. In the midnight blue module, there were three hub genes associated with ion transport: *arahy.19E8GC* encodes cyclic nucleotide-gated ion channels (CNGC15c), *arahy.78PHYW* encodes sodium/calcium exchanger NCL1, and *arahy.AVJ0IE* encodes sodium/metabolite cotransporter BASS1. Two genes (*arahy.SJ8MZN* and *arahy.CRLU4M*) encode mitogen-activated protein kinase kinase kinase 3 (MAPKKK). Two transcription factors, MYB3R-1 and bHLH115, were encoded by *arahy.N15QB0* and *arahy.AJV249*, respectively. An auxin-responsive protein IAA14 encoded by *arahy.QUY0YV* and a NRT1/ PTR FAMILY 4.5 protein encoded by *arahy.BELI5P* were also identified as hub genes associated with salt tolerance in peanut root. A Zinc finger CCCH domain-containing protein 62 encoded by *arahy.J5EU5Y* was also one of the hub salt tolerant genes.

### WGCNA analysis of shoot tissue response to salt stress

In shoot tissue, a total of 11 co-expression modules comprising 11,430 genes were identified based on their similar expression patterns (Fig. 10A). The blue2 module, which comprises 3,912 genes, exhibited a negative correlation with Na^+^ content but a positive correlation with K^+^/ Na^+^ ratio and dry mass. Conversely, the pale turquoise module, containing 2,604 genes, showed a positive correlation with Na^+^ content but a negative correlation with K^+^/ Na^+^ ratio and dry mass, with correlation coefficients ranging between 0.61 and 0.74 (Fig. 10B). The networks for the blue2 and pale turquoise modules were constructed using ten hub genes and their top ten genes with the highest connectivity. The hub genes were visualized as red nodes within the network (Fig. 10C-D).

The hub genes within the blue2 module were consistently down-regulated in shoot tissue and exhibited the lowest expression levels in S1. Conversely, the hub genes in the pale turquoise modules showed a consistent upregulation in shoot tissue, particularly pronounced in S1 and S2 (Fig. 10E). In the blue2 module, *arahy.YB5YRA* encodes arogenate dehydratase/prephenate dehydratase. There were two aquaporins encoded by *arahy.LE80VX* and *arahy.J0HSVP*, and peroxidase 3 encoded by *arahy.ID0B6N*. Beta-amylase is encoded by *arahy.Y6Z03A*, and a photosystem I chlorophyll a/b-binding protein 5 is encoded by *arahy.U5T9YL*. The remaining hub genes in blue2 were *arahy.Q5X08K*, *arahy.2V1UZ8*, *arahy.E3VCX4*, *arahy.HQ5YUL*, which encode kinesin-like protein KIN-7B, acid phosphatase 1, cytochrome P450 77A3, and ABCG32, respectively.

In the pale turquoise module, there were four ion transport related genes. Two hub genes (*arahy.GST19D* and *arahy.4ZG746*) encode sodium/hydrogen exchanger 8 (NHX 8), one gene (*arahy.4S0H6B*) encodes cyclic nucleotide-gated ion channel 2 (CNGC 2), and one gene (*arahy.J8E4BG*) encodes potassium transporter 8. Furthermore, CBL-interacting serine/threonine-protein kinase (CIPK) 11 (encoded by *arahy.N6YX8I*), Phosphatase 2C 51 (encoded by *arahy.8GI8KI*), G-type lectin S-receptor-like serine/threonine-protein kinase (SRK, encoded by *arahy.I9AI1J*), and aldehyde dehydrogenase family 7 (ALDH) member A1 encoded by *arahy.159H25* were also key regulators. *Arahy.FY9BZZ* encodes a late embryogenesis abundant protein LEA5, which plays an important role in stabilizing protein structure and enhancing cell’s water binding capacity.

### Validation of hub genes by qRT-PCR

**Fig. 11:**
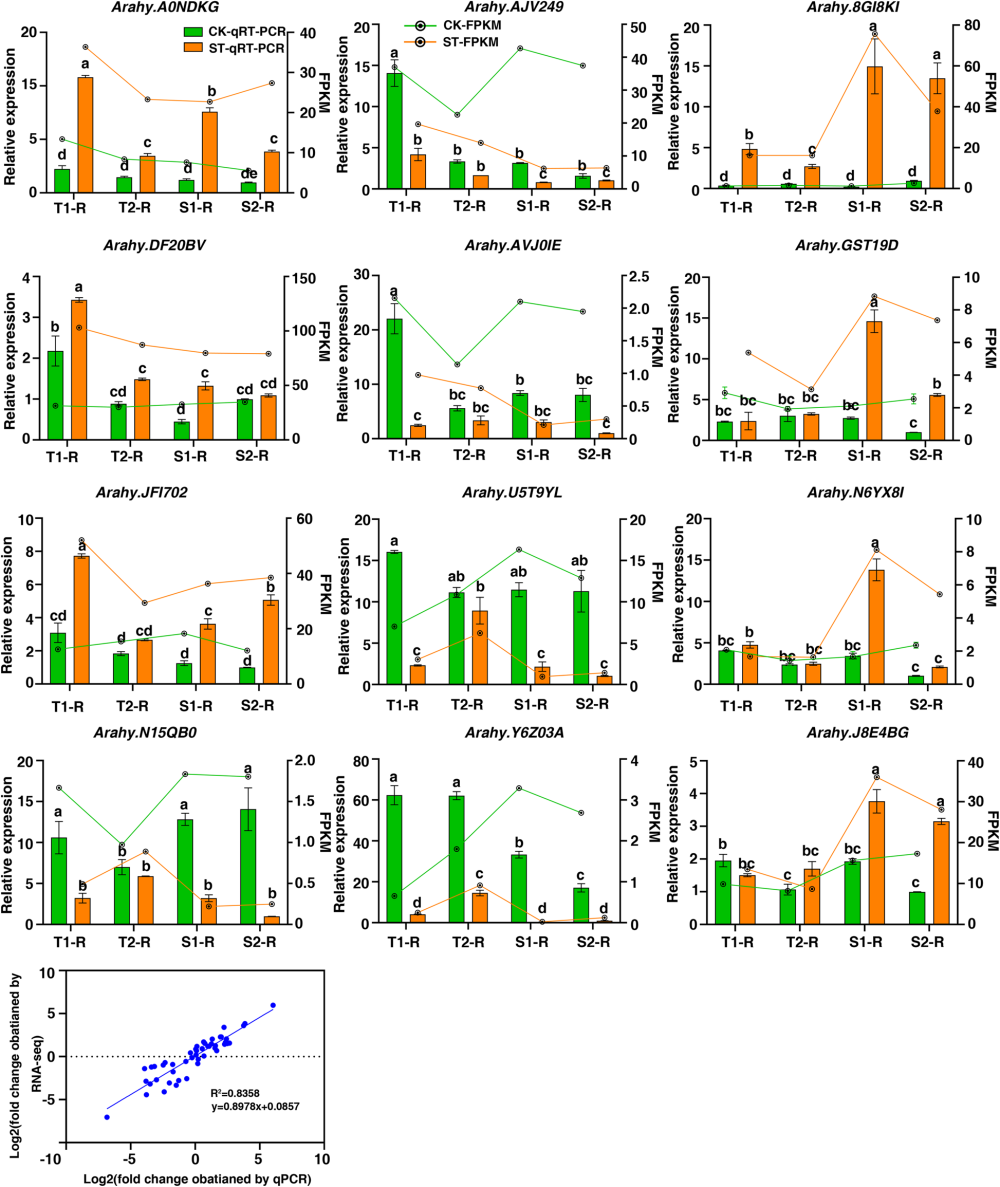
Validation of the hub genes by RT-PCR. Different letters represent significant differences at the 0.05 level. Different lowercase letters indicate a significant difference at *p* ≤ 0.05 (Duncan’s multiple range test)

To validate the reproducibility and authenticity of the RNA-seq data, 12 hub genes with high expression in key modules were selected for qRT-PCR analysis (Fig. 11). qRT-PCR results of all 12 genes were consistent with the expression pattern of RNA-seq data (Figs. 9 and 10). Genes significantly up-regulated in RNA Seq data also exhibited an up-regulation in qPCR, and vice versa. These results also confirmed the reliability of the RNA-Seq data.

## Discussion

Salt tolerance in plants is a complex process influenced by multiple genes. In our study, we utilized RNA-seq and Weighted Gene Co-expression Network Analysis (WGCNA) to investigate the molecular mechanisms underlying the response of root and shoot tissues to salt stress in peanut. Our results indicate that roots are more sensitive to salt stress compared to shoots, with noticeable differences in their biological responses to salt. Through WGCNA analysis, we identified key hub genes responsible for regulating salt tolerance in both peanut root and shoot tissues. In contrast to the findings of Gharaghanipor et al. [38], although the ratio of K^+^/ Na^+^ decreased upon exposure to salt treatment, there were no significant differences among the four peanut genotypes in both root and shoot tissues (Fig. 2G-H). This suggests that Na^+^ exclusion predominantly determines salt sensitivity in peanut similar to chickpeas [39, 40]. Furthermore, the salt-tolerant varieties maintained higher root and shoot growth compared with salt-sensitive varieties, despite similar concentrations of Na^+^ in both root and shoot tissues, consistent with previous studies [39, 40]. T2 exhibited the highest salt tolerance among the four genotypes selected in this study.

Under salt stress, plants employ mechanisms to maintain low cytoplasmic Na^+^ levels [3] and sequester Na^+^ away from photosynthetic tissues. This is primarily achieved through two mechanisms: Na^+^ exclusion from the cell cytosol using plasma membrane Na^+^/H^+^ antiporters (SOS1), and the sequestration of Na^+^/K^+^ into vacuoles via tonoplast Na^+^/H^+^ antiporters (NHXs). SOS1, a crucial component of the salt overly sensitive (SOS) pathway, facilitates Na^+^ efflux from the cytoplasm to the soil [7, 16, 39]. This pathway involves a Ca^2+^-binding protein SOS3 (calcineurin B-like protein (CBL)) interacting with SOS2 (CIPK24) to form a protein kinase complex, subsequently enhancing the expression of SOS1 [16, 39]. In shoot tissues, CBL-interacting serine/threonine-protein kinase (CIPK) 11, encoded by *arahy.N6YX8I*, was identified as one of the hub genes. Apple MdCIPK13 phosphorylated a sucrose transporter MdSUT2.2 to enhance its stability and sucrose transportation activity, thereby improving salt tolerance [41]. NHXs, monovalent ion exchangers, facilitate the movement of Na^+^ or K^+^ to regions of high ion concentration while exchanging with H^+^ across the membrane to maintain local potential conservation [42–44]. Overexpression of NHX genes such as *GmNHX* in soybean and *Arabidopsis* has been shown to enhance salt tolerance [45–47]. In this study, two hub genes (*arahy.GST19D* and *arahy.4ZG746*) encoding sodium/hydrogen exchanger 8 (NHX 8) in shoot tissues exhibited induced expression under salt treatment (Fig. 10E).

Additionally, cyclic nucleotide-gated ion channels (CNGCs) act as non-selective cation channels (NSCCs) and play roles in K^+^ and Na^+^ transport [48]. In the dark slate blue module of root tissue, *arahy.19E8GC* encodes a cyclic nucleotide-gated ion channel (CNGC15c). In shoot tissues, *arahy.4S0H6B* encodes cyclic nucleotide-gated ion channel 2 (CNGC 2), which exhibits a higher degree of K^+^ and Ca^2+^ selectivity compared with Na^+^ in other crops [49, 50]. Furthermore, sodium/calcium exchanger NCL1 and sodium/metabolite cotransporter BASS1 also play important roles in the peanut's response to salt stress.

Maintaining potassium (K^+^) homeostasis is essential for plant survival in high-salt environments and is regulated synergistically by promoting K^+^ influx while inhibiting K^+^ efflux [6, 51]. K^+^ influx is facilitated by plasma membrane (PM) inward K^+^ channels such as HAKs and AKT1 [52]. The KT/HAK/KUP family, which includes K^+^ transporter, high-affinity K^+^ transporter, and K^+^ uptake protein, serves as the primary K^+^ acquisition system in plants, regulating K^+^ uptake and translocation [19]. The function of HAKs has been identified in various crops and plants [6, 53–56]. A hub gene (*arahy.J8E4BG*) encoding potassium transporter 8 (HAK8) may also function as a Na^+^ transporter, as indicated by the negative correlation of the blue2 module with Na^+^ concentration in shoot tissue. Additionally, two aquaporin genes encoded by *arahy.LE80VX* and *arahy.J0HSVP*, involved in transporting water and other small molecules across biological membranes, have been reported to contribute to salt stress tolerance in crops [57, 58].

Mitogen-activated protein kinase (MAPK) cascades serve as crucial components in the response to salt stress, acting as relay systems. Osmotic stress triggers the activation of SnRK2s via an ABA–PYLs–PP2Cs-mediated regulatory module and RAF kinases. Once activated, MPKs and SnRK2s regulate the expression of stress-responsive genes through downstream transcription factors [6]. In our study, two genes (*arahy.SJ8MZN* and *arahy.CRLU4M*) encode mitogen-activated protein kinase kinase kinase 3 (MAPKKK), which plays an essential role in MAPK signal perception, transduction, and amplification [38]. Additionally, phosphatase 2C 51 (encoded by *arahy.8GI8KI*), a component of ABA signal transduction, is known to directly participate in plant salt stress regulation [59, 60]. Furthermore, the hub gene *arahy.ID0B6N* encodes peroxidase 3, which plays a significant role in antioxidant responses and stress tolerance in plants, contributing to salt tolerance in soybean as well [61]. Late embryogenesis abundant (LEA) genes are pivotal in mitigating the effects of active oxygen radicals and preserving cell membrane integrity. These genes are activated in response to water deficit caused by desiccation, cold, or osmotic stress in a wide range of plant species [14]. *Arahy.FY9BZZ* encodes a late embryogenesis abundant protein Lea5, which plays an important role in stabilizing protein structure and enhancing water binding capacity of cells. Beta-amylase is encoded by *arahy.Y6Z03A*, which is the stress-responsive gene and related to accumulation of osmolytes for water and nutrient uptake [6, 13].

The rate of photosynthesis is known to be influenced by salt stress, with photosystem I chlorophyll ab-binding proteins (LHC) playing a crucial role in this process and being widely implicated in plant stress responses. *TaLHC86* has been identified as a beneficial gene for salt tolerance [62]. In our study, we identified a photosystem I chlorophyll a/b-binding protein 5 (encoded by *arahy.U5T9YL*) in the blue2 module of shoot tissue, indicating its involvement in salt tolerance mechanisms. Additionally, several transporters, including a sugar transport protein (STP, *arahy.P6VCNZ*), a phosphate transporter (PHT, *arahy.JFI702*), and NRT1/ PTR FAMILY 4.5 protein (*arahy.BELI5P*), were also found to be associated with salt tolerance. STP proteins play vital roles in monosaccharide absorption in plant tissues or cells, contributing significantly to both plant growth and stress resistance. For instance, the silencing of the *GhSTP18* gene in cotton enhances tolerance to salt stress [63]. Furthermore, a phosphate transporter, PHT4;6, facilitates the selective transport of Pi, crucial for protein N-glycosylation and cell wall biosynthesis, both of which are essential for salt tolerance [64].

In this study, several new salt-tolerant genes in peanuts were identified. *Arahy.J5EU5Y*, encodes a Zinc finger CCCH domain-containing protein 62. Previous research has consistently highlighted the importance of Zinc finger CCCH proteins in plant salt tolerance [65–68]. Additionally, within the dark slate blue module, *arahy.A0NDKG* encodes cytochrome P450, which catalyzes numerous biochemical reactions and plays essential roles in plant growth, development, and secondary metabolism. Moreover, cytochrome P450 has been implicated in the plant's response to salt stress in various species such as *Arabidopsis thaliana* [69, 70], *Medicago truncatula* [71], and *Gossypium hirsutum* [72].

*Arahy.DF20BV* encodes glutathione S-transferase, which plays a crucial role in plant cell detoxification and is closely associated with both biotic and abiotic stresses. Studies have shown that it can enhance salt tolerance in plants like *Arabidopsis* and Poplar [73, 74]. Additionally, *arahy.TN1PNA* and *arahy.5L9AX6* encode fatty acyl-CoA reductase 3 (FAR), which catalyzes the reduction of fatty acyl-CoA or fatty acyl carrier protein substrates, contributing to primary alcohol formation and participating in wax synthesis. Plant cuticular wax, formed in part by FAR activity, acts as a crucial barrier against both biotic and abiotic stresses by preventing water evaporation from the epidermis [75]. *Arahy.CV6J43* encodes MLO-like protein, a plant-specific seven-transmembrane domain protein. Research on the MLO family in rice suggests that environmental stresses can induce changes in H_2_O_2_ levels through MLO-CaM interaction. Generated H_2_O_2_ may function as a signaling molecule, triggering the expression of responsive genes to aid in stress acclimatization [76]. G-type lectin S-receptor-like serine/threonine-protein kinase (SRK, encoded by *arahy.I9AI1J*), belongs to the receptor-like protein kinase (RLK) family, which plays pivotal roles in plant development and responses to adverse environmental conditions, including salt stress regulation [77]. Furthermore, an aldehyde dehydrogenase family 7 (ALDH) member A1 encoded by *arahy.159H25* is associated with salt tolerance in the pale turquoise module. ALDH enzymes irreversibly oxidize aldehyde molecules, providing protection from osmotic stress and generating NAD(P)H. ALDHs are known to be involved in abiotic stress responses in crops [78]. The mechanisms underlying salt tolerance in peanuts are multifaceted and involve complex interactions between various genes and pathways. The potential interaction of hub genes and other genes with highest connectivity should be explored, shedding light on the complexity of regulatory networks involved in salt stress response.

In summary, our investigation revealed that the differential response to salt stress in peanut primarily arises from variations in the expression of genes involved in ion transport, sucrose metabolism, and the transportation of essential nutrients like phosphorus and nitrogen, along with the modulation of MAPK signaling pathways. Significantly, roots are more sensitive to salt stress compared to shoots in peanuts. Furthermore, we identified several hub genes encoding Zinc finger CCCH protein, glutathione S-transferase, fatty acyl-CoA reductase (FAR), MLO-like protein, G-type lectin S-receptor-like serine/threonine-protein kinase (SRK), and aldehyde dehydrogenase (ALDH) enzymes, which are closely associated with peanut salt tolerance. Further research focusing on the functional validation of candidate genes, elucidation of regulatory mechanisms, and epigenetic modifications could provide deeper insights into these mechanisms and facilitate the development of salt-tolerant peanut cultivars to mitigate the impact of soil salinity on crop production.

## Conclusions

This study offers a thorough examination of the root and shoot transcriptome of four distinct peanut genotypes, showcasing their varied responses to salt stress. The comparative analysis of gene expression patterns between salt-sensitive varieties S1 and S2, and salt-tolerant varieties T1 and T2, provides insights into the cultivar-specific molecular mechanisms underlying salt tolerance in peanuts. The hub genes identified through WGCNA analysis predominantly involve ion transport, as well as transporters for sucrose, phosphorus (P), and nitrogen (N), alongside MAPK signaling pathways. These findings represent a valuable resource of candidate genes essential for the development of salt-tolerant peanut cultivars.

### Supplementary Information

Supplementary Material 1.

Supplementary Material 2.

Supplementary Material 3.

Supplementary Material 4.

Supplementary Material 5.

## Data Availability

The datasets used and /or analyzed during the current study are available in the NCBI Bioproject repository under accession number PRJNA1092346 (SRR28497291-SRR28497338).

## Abbreviations

WGCNA: Weighted gene co-expression network analysis
DEGs: Differentially expressed genes
KEGG: Kyoto Encyclopedia of Genes and Genomes
GO: Gene Ontology
MAPK: Mitogen-activated protein kinase
MAPKKK: Mitogen-activated protein kinase kinase kinase 3
FAR: Fatty acyl-CoA reductase
SRK: G-type lectin S-receptor-like serine/threonine-protein kinase
ALDH: Aldehyde dehydrogenase
CNGCs: Cyclic nucleotide-gated ion channels
CBL: Calcineurin B-like protein
CBL: Calcineurin B-like protein
CIPK24: CBL interacting with SOS2
LEA: Late embryogenesis abundant
ME: Module eigengene
